# Comparative and Phylogenetic Analysis of the Chloroplast Genomes of Four Wild Species of the Genus *Prunus*

**DOI:** 10.3390/genes16030239

**Published:** 2025-02-20

**Authors:** Mengfan Cui, Chenxi Liu, Xingling Yang, Mingyu Li, Liqiang Liu, Kai Jia, Wenwen Li

**Affiliations:** College of Horticulture, Xinjiang Agricultural University, Urumqi 830052, China; cuimengfan666@163.com (M.C.); liuchenxi_tz@163.com (C.L.); xjauyangxingling@163.com (X.Y.); limingyu08_17@163.com (M.L.); llq9989@126.com (L.L.); xjau_jk@163.com (K.J.)

**Keywords:** *Prunus*, chloroplast genome, phylogenetic relationship

## Abstract

Background: *Prunus armeniaca*, *Prunus divaricata*, *Prunus tianshanica*, and *Prunus domestica* are valuable ancient tree species that have persisted since the end of the Tertiary period within the Tianshan wild fruit forest. However, the evolutionary relationships among *Prunus* species in the Tianshan wild fruit forest have long posed a challenge. Methods: We sequenced and assembled the chloroplast genomes of *P. armeniaca*, *P. divaricata*, and *P. tianshanica*, and incorporated the chloroplast genome data of *P. domestica* for comparative analysis to elucidate their phylogenetic positions within the genus *Prunus*. Results: The lengths of these chloroplast genomes ranged from 157,395 bp to 158,090 bp, with a total of 130 to 131 genes annotated, comprising 85 to 86 protein-coding genes, 8 rRNA genes, and 35 to 36 tRNA genes. Notably, the four wild *Prunus* species predominantly used high-frequency codons ending in A/U. Additionally, we identified 300 simple repetitive sequences and 166 long repetitive sequences across the four wild *Prunus* species. The mutation sites were mainly found in the non-coding regions, with seven regions of high mutation frequency identified. The phylogenetic tree revealed five branches: subgenus *Armeniaca*, subgenus *Microcerasus*, subgenus *Prunus*, subgenus *Amygdalus*, and subgenus *Cerasus*. The estimated deviation time for the crown group of *Prunus* is roughly 61.41 million years ago. Conclusions: This study provides exhaustive genetic evidence for the classification and systematic relationships of the four wild *Prunus* species and establishes a crucial foundation for subsequent research into the diversity and evolutionary history of the *Prunus* genus.

## 1. Introduction

Ili Tianshan wild fruit forest is situated at the easternmost edge of the Tianshan wild fruit forest belt in Central Asia, exhibiting a belt-like distribution pattern. Its diverse habitat types have established it as a center of biodiversity in Xinjiang, and it has been designated as a key area of terrestrial biodiversity in China [1,2,3]. Since the late Tertiary, the aridification of the climate, coupled with the oscillations of the Quaternary climate, has facilitated the emergence of the Ili Tianshan wild fruit forest as isolated remnants. Important species in this group consist of *P. armeniaca*, *P. divaricata*, *P. domestica*, and *P. tianshanica*. These ancient tree species, which have persisted since the end of the Tertiary, possess significant ecological, economic, and research value [4,5]. The genus *Prunus* sensu lato, which encompasses these species, has long been a subject of taxonomic debate. Taxonomists have historically disagreed on whether *Prunus* should be treated as a single broad genus or divided into multiple segregates, such as *Amygdalus*, *Cerasus*, *Padus*, and *Laurocerasus* [6,7]. This disagreement stems from the morphological, ecological, and genetic diversity observed within the genus. While some taxonomists advocate for a unified *Prunus* based on shared characteristics and evolutionary history, others argue for the recognition of smaller distinct genera to reflect the deep divergences and unique adaptations among its members [8,9]. Since the establishment of molecular systematics, it has been widely used in the study of various plant groups, and since the development of sequencing technology, many scholars have sequenced various subgenera of *Lilium*, constructed molecular phylogenetic trees, and explored the affinities between the subgenera. Phylogenetic analysis aims to elucidate the genetic relationships and common ancestors among species through the construction of phylogenetic trees [10,11]. However, on the basis of the historical diversification of the Tianshan Mountains, the common occurrence of hybridization and assumed rapid radiation makes the phylogenetic status of Xinjiang wild *Prunus* species vague [12].

Following the 1986 publication of chloroplast genome sequences from *Nicotiana tabacum* and *Marchantia polymorpha* [13], there has been a growing emphasis among researchers on elucidating the structural characteristics and variability trends within chloroplast genomes across diverse species. Chloroplast DNA traces its origin from a symbiotic partnership between independent cyanobacteria and eukaryotic organisms, serving a vital function in the process of photosynthesis [14]. In comparison with the nuclear genome, the chloroplast genome, which is inherited maternally, is relatively small, which facilitates sequencing. It has a low nucleotide substitution rate, is haploid, and lacks recombination and mutation [15]. The chloroplast genome typically spans a size range of 120 to 220 kb and features a tetrad organizational structure, comprising a large single-copy (LSC) region, two inverted repeat (IR) regions, and a small single-copy (SSC) region [16]. The structure of the chloroplast genome is stable, with a highly conserved gene composition and a large number of copies, making it easy to extract and purify. A species can be identified by the very different parts of the chloroplast genome [17]. In the study of species, information about the chloroplast genome sequence is very much used, which includes species identification, phylogenetics, molecular evolution, population genetics, and phylogeography [18,19,20,21]. Wang et al. [22] analyzed the chloroplast genomes of six almond species to elucidate the mutational features and evolutionary patterns associated with the almond chloroplast genome. They identified 53 pairs of repetitive sequences and nine highly variable regions, subsequently classifying these six almond species. Their study offers substantial reference value for the identification of almond species. Geng et al. [23] assembled the chloroplast genome of *P. domestica* and carried out a phylogenetic analysis based on the 37 species of the *Prunus* genus that were found in the chloroplast genome sequences of that genus. The results indicated that *P. domestica* is most closely related to *Prunus salicina*. Xue et al. [24] assembled the chloroplast genomes of *Prunus mume*, *P. salicina*, and *P. armeniaca*, which shows that there is a lot of similarity between the three kinds. In comparison with other fruit trees in the Rosaceae family, these three species are more closely related and share a common ancestor. Huang et al. [25] assembled the chloroplast genome of *Prunus zhengheensis*. When compared with seven other chloroplast genomes, it was found that the composition and structure of the chloroplast genome were similar and conserved, although the protein-coding genes *rps18*, *rps12*, *psdF*, *rpl33*, *matK*, and *rbcL* exhibited slight differences. Shen et al. [26] compiled a dataset consisting of 55 chloroplast genomes and executed a divergence time analysis, which elucidated the evolutionary timelines pertinent to the *Cerasus* and its subgenera *Prunus*, a genus within the family Rosaceae, which underwent a separation event during the early Eocene epoch, approximately 51.42 million years ago (95%HPD 40.13–64.32 million years ago). In a comparative analysis of the full sequences of *Prunus Mazzard* and *Prunus avium* “Summit”, Zhao et al. [7] identified the non-coding regions *ndhc-trnV*, *rps12-trnV*, and *rpl32-trnL* as the most variable sequences. Their study highlights that these non-coding regions provide critical information for species identification. By using structural comparisons and phylogenetic analyses of the chloroplast genome to help with our understanding of the evolutionary processes, these studies together provide new information about the identification of species and improve our understanding of the phylogenetic relationships among various varieties or species within the Rosaceae family. Therefore, it is imperative to leverage chloroplast genomic research for the comparative analysis of wild *Prunus* species, thereby elucidating their phylogenetic relationships and temporal divergence patterns.

In this study, the chloroplast genomes of *P. armeniaca*, *P. divaricata*, and *P. tianshanica* were sequenced, assembled, and annotated. The chloroplast genome sequence of *P. domestica* was obtained from NCBI. The aim of this research was to conduct a comprehensive comparison and analysis of these four wild species of the genus *Prunus*. By doing so, a phylogenetic tree was constructed to clarify the phylogenetic relationships among these species within the *Prunus* genus. It was hypothesized that there were significant differences and patterns in the chloroplast genomes of these species of the genus *Prunus* that could be used to understand their phylogenetic relationships and inform related aspects of their identification, classification, and breeding. The results of this study are expected to provide valuable theoretical insights for the identification, phylogenetic classification, and breeding of *Prunus* species.

## 2. Materials and Methods

### 2.1. Plant Material, DNA Extraction, and Sequencing

There was a total of 24 chloroplast genomes, of which 21 were obtained from GenBank (www.ncbi.nlm.nih.gov/genbank, accessed on 15 October 2024), as detailed in Supplementary Table S1. Additionally, we sequenced and assembled the chloroplast genomes of three wild *Prunus* species: *P. armeniaca*, *P. divaricata*, and *P. tianshanica*. Fresh young leaves were collected at the following locations: Almale Township, Xinyuan County, Ili Kazakh Autonomous Prefecture, Xinjiang, China (83°36′27″ E, 43°22′36″ N, 1465.2 m); Ili Botanical Garden, the Xinjiang Institute of Ecology and Geography, the Chinese Academy of Sciences; Xiaoxigou, Huocheng County, Ili Kazakh Autonomous Prefecture, Xinjiang, China (80°49′55″ E, 44°26′12″ N, 1240.1 m); Maoliugou, Beishan Mountain, Tekesi County, Ili Kazakh Autonomous Prefecture, Xinjiang, China (81°57′37″ E, 43°15′21″ N, 1296.2 m). The voucher specimens were deposited in the herbarium of Xinjiang Agricultural University with the voucher numbers 1630 (*P. armeniaca*), 77 (*P. divaricata*), and 56 (*P. tianshanica*), respectively.

The extraction of total DNA was carried out using a plant genomic DNA kit (TIANGEN, Beijing, China). The concentration and quality of the DNA samples were then assessed using a NanoDrop 2000 micro-spectrophotometer (Wilmington, DE, USA) and 1% agarose gel electrophoresis, respectively. The extracted DNA was subsequently fragmented and end-repaired to prepare a genomic library. Following this, the library construction was succeeded by high-throughput sequencing using Illumina HiSeq [27]. Library preparation and sequencing services were expertly conducted by Wuhan Benagen Technology Co., Ltd., Wuhan, China.

### 2.2. Genome Assembly and Annotation

The chloroplast genome was assembled using GetOrganelle v.1.7.5, a process that was made possible by the clean data obtained from sequencing [28]. The complete chloroplast genome was reconstructed through a manual refinement process, leveraging the entire chloroplast genome of *P. armeniaca* (KY101151.1) as a reference sequence. This methodology was executed using the Geneious Prime online software platform (http://www.geneious.com/, accessed on 18 October 2024) [29]. Following assembly, the chloroplast genome was annotated using the Geseq online tool (https://chlorobox.mpimp-golm.mpg.de/geseq.html, accessed on 18 October 2024) [30]. The chloroplast genome was subsequently mapped using Chloroplot online software (https://irscope.shinyapps.io/Chloroplot/, accessed on 18 October 2024) [31].

### 2.3. A Comparative Examination of the Chloroplast Genomic Sequences Among Four Wild Species Within the Genus Prunus

#### 2.3.1. Codon Preference Analysis

The computation of synonymous codon usage frequency for each coding amino acid in the chloroplast genomes of four wild *Prunus* species was executed using CodonW version 1.4.4 software [32]. The relative synonymous codon usage (RSCU) is determined by the comparison of the frequency of a specific codon within a coding sequence to its expectation frequency among a family of synonymous codons. RSCU values near 1.0 denote a lack of codon bias, indicating that all synonymous codons are used with equal frequency. Conversely, RSCU values exceeding 1.0 indicate a codon bias, suggesting that a particular synonymous codon is favored and used more frequently than the average, thus deviating from the general usage pattern [33].

#### 2.3.2. IR Boundary Analysis

Regions that are inverted repeat (IR) show a high degree of conservation among chloroplast genes; however, the main mechanisms causing the size of the genome-wide change in angiosperm chloroplasts are thought to be the expansion and contraction of IR boundaries. We used the online software IRscope (https://irscope.shinyapps.io/irapp, accessed on 20 October 2024) to visualize the expansion and contraction of the IR region over the whole chloroplast genome of four wild *Prunus* species [34].

#### 2.3.3. Repeat Sequence Analysis and SSRs

Simple sequence repeats (SSRs) were analyzed within the chloroplast gene sequences of four wild *Prunus* species by employing the Perl script MISA [35]. The following were the cutoff values for repeat units: 10 for mononucleotide, 5 for dinucleotide, 4 for trinucleotide, and 3 for tetranucleotide repeats, pentanucleotide, and hexanucleotides. We conducted a systematic search for diverse dispersed repeat sequences, including direct, complementary, palindromic, and inverted repeats, using the REPute online computational tool (https://bibiserv.cebitec.uni-bielefeld.de/reputer, accessed on 25 October 2024). To ascertain the types of repetitive sequence present within the chloroplast genomes of the four distinct wild *Prunus* species, the analytical framework was established with a specified minimum sequence length of 30 nucleotides and a Hamming distance of 3, facilitating the detection of sequences that are nearly identical yet not identical [36].

#### 2.3.4. Full Sequence Alignment and Nucleotide Diversity

We used the chloroplast genome of *P. divaricata* as a reference and employed mVISTA in Shuffle-LAGAN mode to conduct a comprehensive sequence comparison of the chloroplast genomes among four wild *Prunus* species [37]. Chloroplast genomes of these four wild *Prunus* species were aligned using MAFFT v. 7.505 software [38]. Subsequently, the nucleotide diversity (Pi) for the chloroplast genomes was quantified using DnaSP v.6.12.03 software [39], employing a window length and step size of 600 bp and 200 bp, respectively.

### 2.4. Phylogenetic Analysis

To achieve a deeper insight into the evolutionary relationships among four **distinct** wild *Prunus* species and their related genera and species, Bayesian inference (BI) and maximum likelihood (ML) methodologies were used to generate the phylogenetic tree [40]. Sequence alignment was conducted using MAFFT v.7.505, with *Malus sieversii* and *Crataegus songarica* serving as outgroups. For the building of ML trees, ModelFinder was used to identify the appropriate model, which was determined to be TVM. The analysis was conducted using IQ-TREE version 2.2, incorporating a bootstrap value of 1000 to assess the statistical support for the inferred phylogenetic relationships [41]. Additionally, Bayesian inference trees were constructed using MrBayes v.3.1.2 with the GTR model, and the Markov chain Monte Carlo (MCMC) algorithm was performed for 1,000,000 generations, with trees being sampled every 100 generations. To test the convergence of the parallel runs, we compared the average standard deviation of the split frequency, which was found to be below 0.01. The remaining 25% of the created trees were used to build the majority rule consensus tree, after excluding the top 25% of the trees [42].

### 2.5. Divergence Time Estimation

Using the online platform TimeTree (http://www.timetree.org/, accessed on 29 October 2024), fossil calibration points for various species were identified. As a result of this search, three calibration points for fossil divergence were established, marking the divergence time between *M. sieversii* and *Prunus pseudocerasus* set at 34.4–67.2 Mya; the divergence time between *P. mume* and *Prunus humilis* set at 33.7–61.5 Mya; and the divergence time between *Prunus cerasus* and Prunus fruticosa also set at 33.7–61.5 Mya [43]. Differentiation times were estimated using the MCMCTREE program within PAML v. 4.9 software. The ML tree topology of *Prunus* was employed to calibrate the differentiation time nodes, using the GTR nucleotide model. The MCMC chain length was set to 1,010,000, with sampling occurring once every 10 runs. The first 10,000 drawings were discarded as burn-ins, resulting in a total of 100,000 samples collected for analysis. Using FigTree v.1.4.2 software, the final results were reviewed and modified [44].

## 3. Results

### 3.1. General Characteristics of the Chloroplast Genome

**Figure 1:**
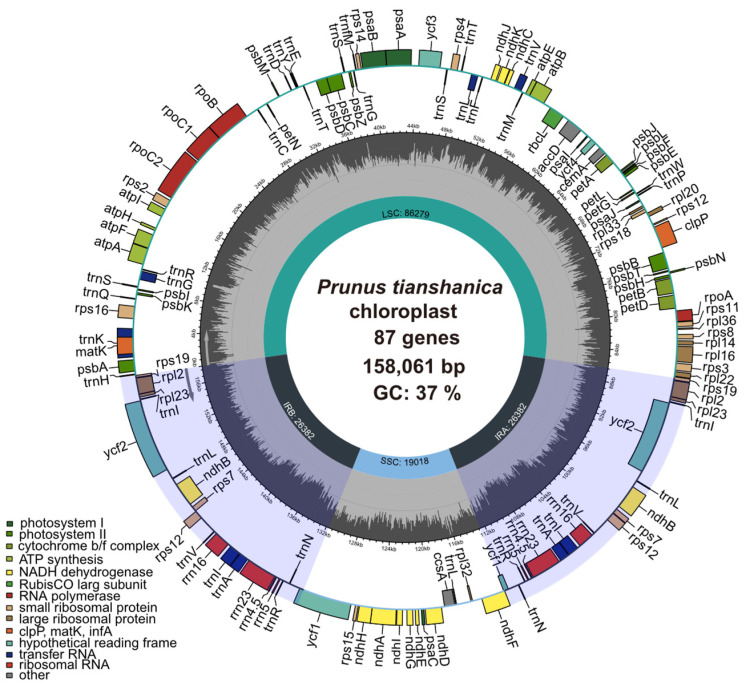

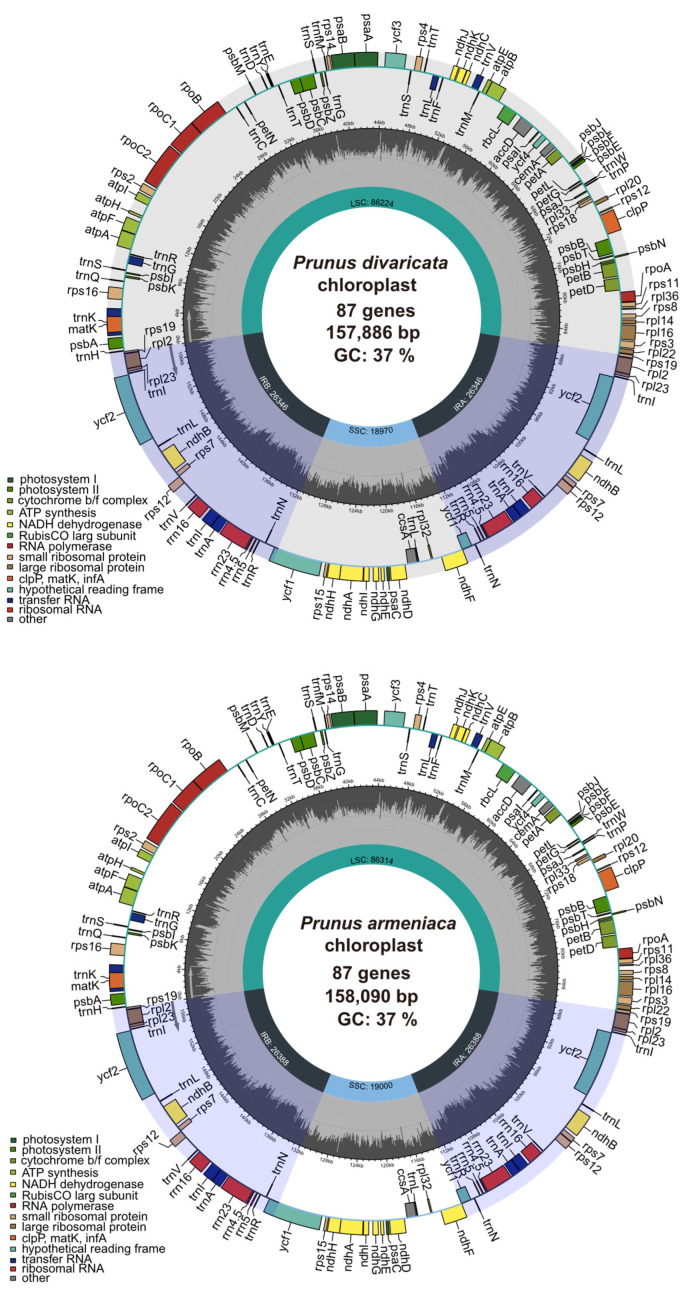
Chloroplast genome map of three wild species of the genus *Prunus*. The central focus of the illustration presents detailed genomic data, including the total length of the genome, GC content, and the number of genes. Genes shown outside the circle are transcribed in the counter counterclockwise direction, while those inside the circle are transcribed in the clockwise direction. Gene with different functions are represented in various colors. The light grey area indicates the GC content, while the dark grey area represents the non-GC content.

The chloroplast genomes of *P. armeniaca*, *P. divaricata*, and *P. tianshanica* were sequenced using Illumina HiSeq (Illumina Inc., San Diego, CA, USA), which produced clean data. The sizes of the chloroplast genomes of these four wild *Prunus* species ranged from 157,395 to 158,090 bp, exhibiting a typically circular structure (Figure 1). The sequence of shortest length was found to be 157,395 bp in *P. domestica*, while the longest was 158,090 bp in *P. armeniaca*. Post-optimization, the efficiencies for both Q20 and Q30 metrics surpassed 93.23%. The chloroplast genomes of the four wild *Prunus* species displayed a characteristic tetrameric configuration, comprising an LSC region (85,744 to 86,314 bp), an SSC region (18,949 to 19,018 bp), and two IR regions (26,346 to 26,388 bp). The GC contents of the chloroplast genomes for *P. armeniaca*, *P. divaricata*, *P. tianshanica*, and *P. domestica* were found to be 36.72%, 36.74%, 36.73%, and 36.76%, respectively. It is noteworthy that the GC content was significantly less than the AT content, which is in accordance with the AT abundance characteristic of chloroplast genomes. Additionally, the examination of the IR region showed a significant rise in GC content, increasing from 42.56% to 42.63%, in comparison with the LSC (34.53% to 34.56%) and SSC (30.42% to 30.47%) regions (Table 1). These results indicate that the IR region exhibits greater stability compared with the SSC and LSC regions. Four wild *Prunus* species were used in the current investigation to annotate the chloroplast genomes of the species. A total of 130 genes were identified in wild *P. domestica*, comprising 85 protein-coding genes (PCGs), 37 tRNAs, and 8 rRNAs. The remaining three wild *Prunus* species were found to possess 131 genes apiece, encompassing 86 protein-coding genes (PCGs), 37 transfer RNAs (tRNAs), and 8 ribosomal RNAs (rRNAs) each. While the gene content of the four wild *Prunus* species was largely similar, some differences were noted. All species were annotated with the pseudogene *rps19*, except for *P. domestica*, which lacked this gene. Additionally, *P. tianshanica* possessed a unique pseudogene, *ycf1*, while both *P. divaricata* and *P. tianshanica* had duplicated the *trnS-GCU* gene. The *trnS-GGA* gene was unique to *P. armeniaca* and *P. domestica*. The categorization of gene functions within the chloroplast genome is delineated into four primary classes: self-replicating genes, those involved in photosynthesis, genes with unspecified roles, and genes of unknown function. In the analyzed set of sequences, a total of 17 repetitive sequences were identified, of which 15 sequences featured a single intron (comprising *ndhA*, *ndhB*, *petB*, *petD*, *atpF*, *rpl16*, *rpl2*, *rps16*, *rpoC*, *trnA-UGC*, *trnG-GCC*, *trnI-GAU*, *trnK-UUU*, *trnL-UAA*, *trnV-UAC*). Notably, only three sequences, *rps12*, *clpP*, and *ycf3*, harbored two introns (Table 2).

### 3.2. Codon Usage Preference Analysis

**Figure 2:**
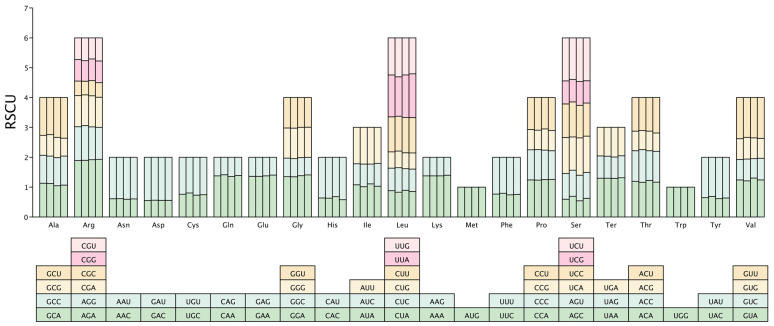
The relative synonymous codons of the four wild *Prunus* species are represented by histograms. For each codon block that specifies a distinct amino acid, the upper columns delineate the cumulative relative synonymous codon usage (RSCU) values across the 20 amino acids. The four columns are arranged as follows: *P. armeniaca*, *P. divaricata*, *P. domestica*, *P. tianshanica*.

**Figure 3:**
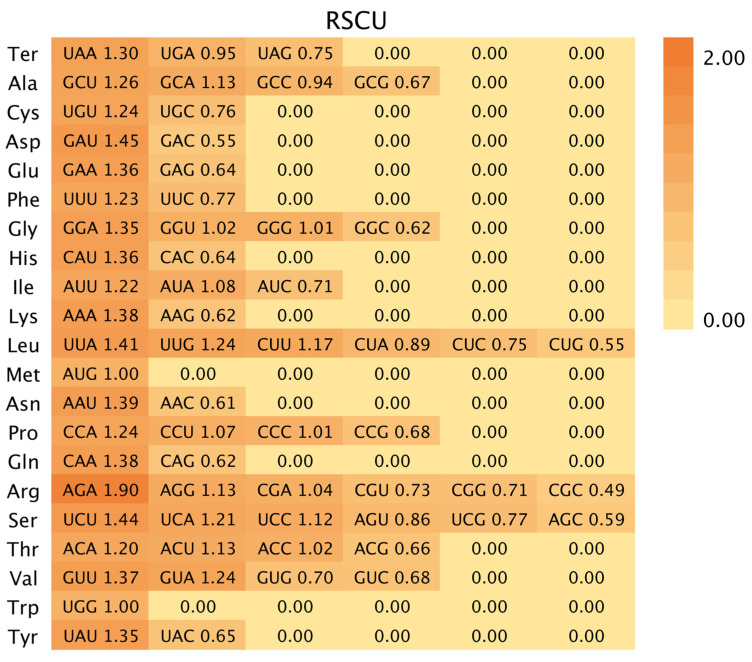
The codon usage frequencies within the chloroplast genomic sequences of four wild Prunus species investigated. The hues depicted in the figure correspond to the RSCU values, with codon usage frequencies escalating progressively from light yellow to orange.

Codon bias has evolved in organisms over extended evolutionary periods and involves a complex array of mechanisms. Chloroplast genomes of *P. armeniaca*, *P. divaricata*, *P. tianshanica*, and *P. domestica* contained 52,696, 52,628, 52,687, and 52,465 codons, respectively. The chloroplast genome compositions of the four investigated *Prunus* species encompassed a collective total of 61 synonymous codons responsible for the synthesis of 20 distinct amino acids, as illustrated in Figure 2. Among these amino acids, leucine (Leu) was the most prevalent, accounting for 5039 to 5464 codons, which represented 9.57% to 10.33% of the total. Additionally, serine (Ser) was encoded by 3196 to 3287 codons (8.78% to 9.35%), and arginine (Arg) was represented by 4625 to 4928 codons (6.07% to 6.27%), while tryptophan (Trp) was the least frequently used, appearing in 622 to 693 codons (1.19% to 1.31%) (Supplementary Table S2). Of all the codons, the most often used was AGA, whereas the least used was CGC (Figure 3). A comprehensive analysis revealed the identification of 34 high-frequency codons (with a relative synthesis codon usage greater than 1) across the chloroplast genomes of four *Prunus* species. Among these, 28 codons (constituting 82.4% of the total) concluded with adenine (A) or uracil (U) bases, highlighting a predominant termination pattern of high-frequency codons with A/U bases within these species’ chloroplast genomes. Conversely, 27 low-frequency codons (RSCU < 1) were identified, with 24 (88.9%) ending in G/C, suggesting a tendency for low-frequency codons in these chloroplast genomes to conclude with G/C. It is evident that the codon usage preferences exhibited by the chloroplast genomes of the four wild *Prunus* species are largely similar, exhibiting minimal variation.

### 3.3. IR Boundary Analysis

**Figure 4:**
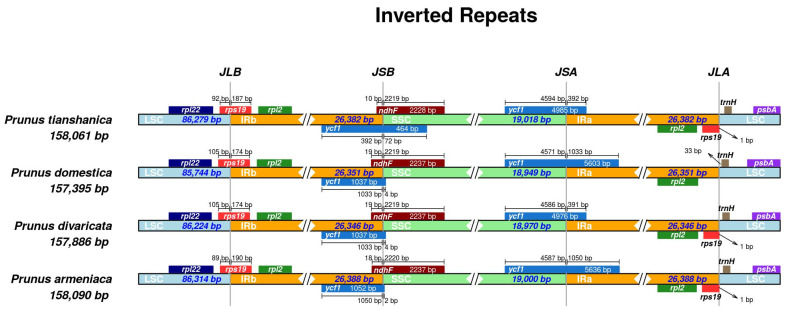
Comparative analysis of IR boundaries in the chloroplast genomes of four wild Prunus species. Delineation of IR regions in relation to SSC and LSC segments. Intergenic distances between boundary termini and adjacent genes are indicated in bp along the primary axis.

The modulation of the IR region’s size through contraction and expansion processes is foundational to the dynamics governing chloroplast genome architecture, acting as the primary factor influencing its size variability. Through the application of IRscope technology, we meticulously recorded the dynamic changes in chloroplast genome sizes across the four distinct wild *Prunus* species (Figure 4). An examination of the chloroplast genome boundaries in four wild species within the genus *Prunus* revealed that the lengths of their IRs were relatively conserved, demonstrating no substantial contraction or expansion. A limited number of base pairs exhibited negligible differences in comparison with the 69 to 570 bp length variations observed in the SSC and LSC regions. The chloroplast genomes of four wild species from the genus *Prunus* featured four genes *rps19*, *ycf1*, *ndhF*, and *trnH* located at the LSC-IRb, SSC-IRb, SSC-IRa, and LSC-IRa boundaries, with *rps19*, *ycf1*, and *ndhF* spanning the LSC-IRb and SSC-IRb regions. The *rps19* gene fragments uniformly exhibited a length of 279 base pairs, whereas the expansions within the *rps19* gene displayed minor variations in their length, spanning from 89 to 105 base pairs within the LSC region. The delineation of the boundary between the IRb and SSC regions was achieved through analysis of the *ycf1* and *ndhF* genes. Specifically, the *ycf1* gene was found to predominantly occupy the IRb region, whereas the *ndhF* gene was primarily located within the SSC region. The full-length sequences of these genes exhibited variability, ranging from 464 to 1052 base pairs for the *ycf1* gene and from 2228 to 2237 base pairs for the *ndhF* gene, respectively.

### 3.4. Sequence Analysis of Repetitive Sequences and SSRs

**Figure 5:**
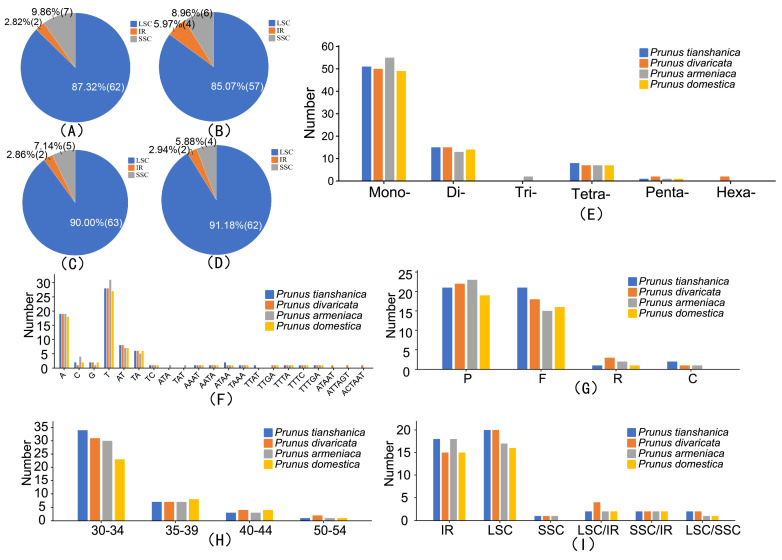
Repetitive sequences found in the genomes of four wild species in the genus *Prunus*. (**A**) represents the distribution of SSRs in different copy regions of *P. armeniaca*; (**B**) shows the distribution of SSRs in different copy regions of *P. divaricata*; (**C**) illustrates the distribution of SSRs in different copy regions of *P. tianshanica*; (**D**) depicts the distribution of SSRs in different copy regions of *P. domestica*; (**E**) presents the classification of six types of SSRs; (**F**) shows the classification of different SSR repeat unit types; (**G**) frequency of each type by length; (**H**) frequency of each type by length; (**I**) frequency of each type by length.

A total of 300 SSRs were detected in the chloroplast genomes of four wild *Prunus* species. The largest amount of mononucleotide repeats was detected with 205 repeats (68.33%), followed by dinucleotide repeats (57 repeats, 19.00%) and tetranucleotide repetitions (29 repeats, 9.67%). Trinucleotide repetitions were found in 2, 5, and 2 instances, respectively, in pentanucleotide repeats, pentanucleotides, and hexanucleotide repeats (Figure 5E). The analysis of SSR distribution across the four replicate regions in four distinct wild *Prunus* species demonstrated a uniform pattern. In the analyzed dataset, the LSC region demonstrated the highest frequency of SSRs, with percentages spanning from 85.07% to 91.18%. This was succeeded by the SSC region, which contributed to the SSR composition with percentages varying from 5.88% to 9.86%. The prevalence of SSRs within the IRa and IRb was noted to be relatively minimal and uniformly distributed, spanning a spectrum from 2.82% to 5.97% (Figure 5A–D). In the chloroplast genomes of four distinct wild *Prunus* species, the SSR sequences predominantly featured adenine (A) and thymine (T) nucleotides, with more than 50% of the SSR sequences being exclusively composed of these two bases. Wild *Prunus* species *P. armeniaca* had the highest single nucleotide repeat rate (70.51%), followed by *P. domestica* (69.01%), *P. tianshanica* (68.00%), and *P. divaricata* (65.79%). The most common SSR type in *P. armeniaca* was characterized by a single nucleotide repeat sequence, specifically T with 31 repeats. Similarly, *P. tianshanica* and *P. divaricata* both exhibited 28 repeats, while *P. domestica* had 27 repeats, respectively (Figure 5F).

Four wild *Prunus* species were the genomes of which were chloroplasts, and the type, amount, and length of the large repeat sequences were analyzed. The analysis produced a total of 166 significant repeat sequences, comprising 70 forward repeats, 85 palindromic repeats, 4 complementary repeats, and 7 reverse repeats. Among the four *Prunus* species, *P. armeniaca* exhibited the least number of forward repeats (15 repeats), while *P. tianshanica*, *P. divaricata*, and *P. domestica* had, respectively, 21, 18, and 16 forward repeats. *P. domestica* exhibited the lowest number of palindromic repeat sequences (19 repeats), while *P. armeniaca*, *P. divaricata*, and *P. tianshanica* had, respectively, 23, 22, and 21 palindromic repeat sequences (Figure 5G). *P. domestica* did not have complementary repeats. Among these large repeat sequences, the repetitive sequences were concentrated in 30–34 bp, 118 of them accounted for 71.08%, and none of the four wild *Prunus* species had repetitive sequences in 45~49 bp (Figure 5H). The largest proportion of sequences was observed within the LSC region, succeeded by the IR region. Furthermore, certain repetitive sequences were observed to be conserved across the SSC, IR, and LSC regions (Figure 5A–D).

### 3.5. Comparative Analysis of Chloroplast Genome Sequences and Assessment of Nucleotide Diversity

**Figure 6:**
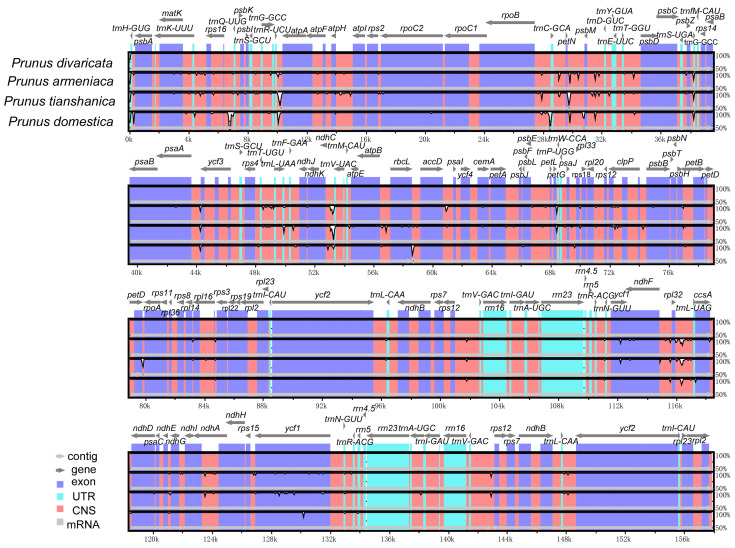
Subsequent sequence identity plots depict the chloroplast genome sequences of four wild species within the genus *Prunus*. The orientation of the genes is indicated by the grey arrows; non-coding sequences are denoted by red bars, exons by purple bars, and introns by blue bars. The vertical scales indicate percentage identity in the range of 50% to 100%.

**Figure 7:**
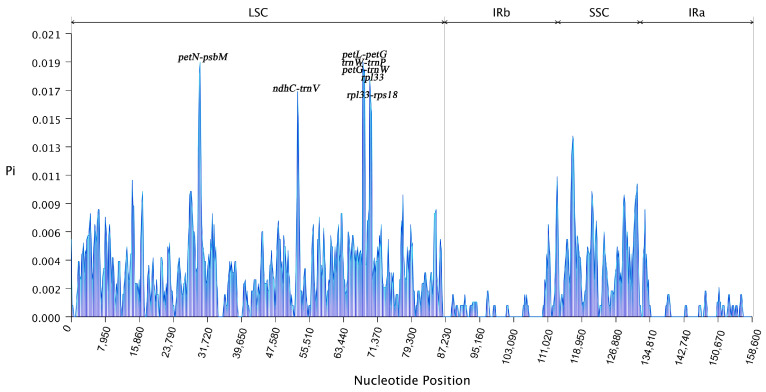
Sliding window analysis of the four wild species of Prunus that belong to the chloroplast genomes of Pi. Each window has its nucleotide diversity shown by the *y*-axis, whereas each window has a position represented by the *x*-axis of the sliding window.3.6. Phylogenetic analysis and time estimation.

The comparison of complete chloroplast genomes enabled a more accurate examination of sequence variations among the four wild *Prunus* species. The results showed that sequence comparison revealed a low degree of variation in the chloroplast genes of the four wild species of the genus *Prunus* (Figure 6). The focus was on non-coding regions, where observed variability was concentrated. The nucleotide diversity parameters for the four wild species within the *Prunus* genus indicated minimal variation in the LSC region, with Pi values ranging from 0 to 0.019 (Figure 7). Seven regions of the chloroplast genes of the four wild species of the genus *Prunus* were identified as highly variable in the LSC region (Pi > 0.015), namely *petN-psbM*, *ndhC-trnV*, *petL-petG*, *trnW-trnP*, *petG-trnW*, *rpl33*, *rpl33-rps18*.

**Figure 8:**
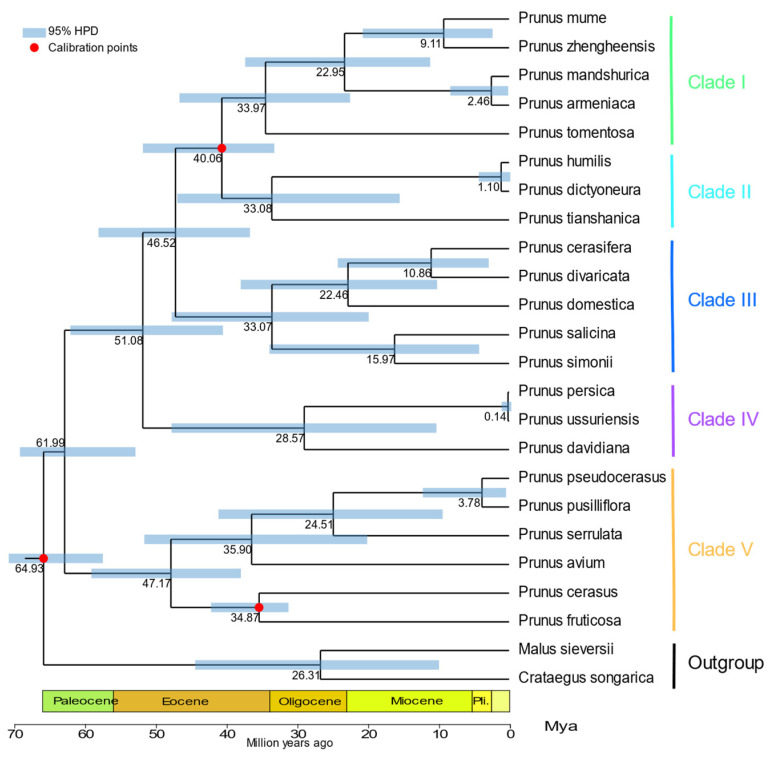
Estimation of differentiation time based on the ML tree of species of the genus *Prunus*. The three calibration points are shown by the red dot, and the 95% highest posterior density is shown by the blue line.

The elucidation of chloroplast genome sequences is fundamental for uncovering the complex evolutionary phylogenetic relationships among plant species and for enhancing the systematic classification within plant taxonomy. BI and ML approaches were employed to construct phylogenetic trees, which provided insights into the evolutionary positioning of wild *Prunus* species within the comprehensive *Prunus* genus. The results of this study indicate that the two employed approaches yielded identical phylogenetic trees with a high support rate, demonstrating the stability of the constructed trees (Supplementary Figure S1). The five branches that make up the evolutionary tree are shown in Figure 8. Clade I exhibits a relatively complex composition, comprising subg. *Armeniaca* (including *P. mume*, *P. zhengheensis*, *Prunus mandshurica*, and *P. armeniaca*) and subg. *Microcerasus*, represented by *Prunus tomentosa*. Notably, *P. armeniaca* and *P. mandshurica* form one branch. Clade II is categorized under subg. *Microcerasus*, with *P. tianshanica* identified as sister to *Prunus dictyoneura* and *P. humilis*. Clade III encompasses all species belonging to subg. *Prunus*, with *Prunus* cerasifera and *P. divaricata* forming a clade that is sister to *P. domestica*. Clade IV is entirely composed of species from subg. *Amygdalus*, while Clade V consists solely of species from subg. *Cerasus*.

The findings suggest that the estimated emergence of the crown group of *Prunus* occurred around 64.93 million years ago (Mya), with a 95% highest posterior density (HPD) interval ranging from 56.98 to 69.45 Mya. In Clade I, the estimated crown group age for the subgenus *Armeniaca* was 22.95 million years ago (95%HPD 11.33–36.48 million years ago), whereas the estimated crown group age for the subgenus *Cerasus* was 33.08 million years ago (95%HPD 15.59–45.95 million years ago). In Clade III, the time estimation for *P. domestica*, derived from *P. cerasifera* and *P. divaricata*, was 22.46 Mya (95% HPD: 10.36–37.09 Mya). The branch time estimation for Clade IV of subg. *Amygdalus* was 28.57 Mya (95% HPD: 9.59–40.21 Mya), and the crown cluster time estimation for Clade V of subg. *Cerasus* was 47.17 Mya (95% HPD: 37.13–57.93 Mya) (Figure 8).

## 4. Discussion

### 4.1. Chloroplast Genome Characteristics of Four Wild Prunus Species

An analysis was conducted on the chloroplast genomes of four wild *Prunus* species for the purposes of comparison, phylogenetic analysis, and divergence time estimation. These species belonged to the same genus *Prunus* group [44]. The chloroplast genomes of these four wild *Prunus* species exhibited both structural and length similarities, all displaying a typical tetrameric configuration. The genome size ranged from 157,395 to 158,090 bp, and the number of genes varied from 130 to 131, consistent with the previously reported chloroplast genome of *P. domestica* [23]. All GC contents in the four chloroplast genomes were similar, ranging from 36.72% to 36.76%. Notably, the analysis revealed that the GC concentration within the IR area surpassed that of both the LSC (34.53–34.56%) and SSC (30.42–30.47%) regions, with values ranging from 42.56% to 42.63%. This elevated GC content is hypothetical to contribute to the stability of the genomes, and the conservatism observed in the IR region may be linked to its GC content. The increased GC content detected in the rRNA genes inside the IR area may be the cause of the observed discrepancies. In conclusion, the chloroplast genomes of the four wild *Prunus* species displayed a high degree of similarity in terms of GC content, the number and location of introns, genotype, and length.

### 4.2. Codon Usage Bias of Four Wild Prunus Species

Codon preference, a complex biological phenomenon, is shaped by a multitude of factors, including species origin, mutation dynamics, and evolutionary history. The examination of codon usage patterns is indispensable for elucidating the evolutionary forces acting upon wild *Prunus* species and for advancing genetic research in this domain [45]. In the chloroplast genome, of the 20 amino acids, all are encoded by two or more codons, except for MET and TRP, each of which is encoded by a single codon. RSCUs are used as a direct indicator to indicate the preference for codon usage. Notably, the four wild *Prunus* species were found to have codons ending in A/U, which was the reason for the high-frequency codon use bias in the chloroplast genomes. This pattern is consistent with observations in numerous angiosperms, including *Malus baccata* [46], *Chrysanthemum morifolium* [47], and *Paeonia ludlowii* [48], which may exhibit similar patterns of codon usage. The codon usage bias in plants is primarily influenced by mutational pressure, implying that closely related species may exhibit similar codon usage patterns. This similarity can serve as a valuable reference for analyzing phylogenetic relationships [49].

### 4.3. IR Boundary Analysis of the Chloroplast Genome of Four Wild Prunus Species

In angiosperms, chloroplast genomes exhibit a notable level of conservation. Nonetheless, the primary mechanism driving variations in their size is hypothesized to be the fluctuations at the interfaces between the IR and the SSC [50]. For instance, the chloroplast genome of *P. avium* lacked the *rps19* gene, while the genome of *Prunus scopulorum* underwent expansion or contraction in the IR region. Additionally, a duplication of the *rps19* gene was observed in the IR region of *P. humilis* [26]. Through comparative analysis of the chloroplast genomes of four wild *Prunus* species, we detected a contraction or expansion event in the *ycf1* gene at the SSC/IR boundary and a deletion of the *rsp19* gene at the IRa/LSC boundary in the chloroplast genome of *P. domestica*. These dynamic changes in the IR region may be associated with the adaptive evolution of species, particularly during environmental shifts or species differentiation [51].

### 4.4. Repetitive Sequences and SSRs in the Chloroplast Genome

Repetitive sequences are fundamental to the structure, evolution, and functionality of plant genomes [52]. Comparative studies across various chloroplast genomes have highlighted that these sequences significantly influence gene insertions, deletions, and substitutions [53]. SSRs are widely used as molecular markers in population genetics, population structure analysis, and biodiversity assessments due to their high degree of polymorphism and the ease with which they can be identified [54,55]. Moreover, long repetitive sequences are crucial for species’ adaptive evolution, contributing to genomic organization differences through structural variations induced by their extended segments. In this study, we identified 300 SSRs across the chloroplast genomes of four wild *Prunus species*, mainly comprising mononucleotide A/T repeats. The abundance of these repeats in chloroplast genomes may stem from their functional roles and adaptive importance in biological evolution [56].

### 4.5. Comparative Sequence Analysis and Nucleotide Diversity Analysis of the Chloroplast Genome

Full sequence comparison is crucial for species identification analysis, as it involves the comparison of chloroplast genomes across various species or individuals to identify similarities and differences. Areas that are very varied can be used as useful molecular markers [57]. Our study revealed that differences were more pronounced in non-coding regions, while the IR region exhibited greater conservation compared with the LSC and SSC regions. Seven highly variable regions were accurately identified in the chloroplast genomes of the four wild *Prunus* species, including *rpl33*, and six gene spacer regions—*petN-psbM*, *ndhC-trnV*, *petL-petG*, *trnW-trnP*, *petG-trnW*, and *rpl33-rps18*—based on Pi values. These very different locations found in this work might be used as molecular markers for the identification of wild *Prunus* species.

### 4.6. Phylogenetic Analysis and Time Estimation

Wild *Prunus* species are significant in the Tianshan Mountains, and the genetic evolution of species of the genus *Prunus* has been a topic of significant debate for a long time. In our study, the phylogenetic tree of *Prunus* was classified into five branches: subg. *Armeniaca*, subg. *Microcerasus*, subg. *Prunus*, subg. *Amygdalus*, and subg. *Cerasus*. This clustering exhibited a high level of support, with most nodes demonstrating 100% support. Notably, *P. armeniaca* and *P. mandshurica* were identified as a single branch, with *P. armeniaca* showing a closer relationship to *P. mandshurica* than to *P. mume* or *P. zhengheensis*. Zhang et al. [56] used ITS genes to conduct a phylogenetic analysis of the subgenus *Armeniaca*, yielding findings suggesting a greater phylogenetic distance between *P. armeniaca* and *P. mume*. We found that *P. tomentosa* was distinct from and phylogenetically distant to subgenus *Cerasus*, and was clearly separated from other species in subgenus *Microcerasus.* Notably, *P. tomentosa* was more closely related to subgenus *Armeniaca*. According to Yu Dejun’s [58] definition, the genus *Cerasus* was divided into subg. *Cerasus* and subg. *Microcerasus*, with *P. tomentosa*, *P. tianshanica*, *P. dictyoneura*, and *P. humilis* categorized under subg. *Microcerasus*. Zhao et al. [7] performed a phylogenetic analysis on 20 species of *Prunus* using chloroplast genomic data, which indicated that *P. tomentosa* and *P. pedunculata* formed a separate branch, yet both were classified under subgenus *Prunus*. However, our study posits that *P. tomentosa* is more closely aligned with subgenus *Armeniaca* and should therefore be classified as such. Wan et al. [57] observed that species within subgenus *Microcerasus* and subgenus *Cerasus* segregated into distinct branches, demonstrating closer relationships between subgenus *Microcerasus* and subgenus *Amygdalus*. Chen [8] noted that species in subgenus *Microcerasus* tended to cluster with those in subgenus *Amygdalus*, subgenus *Armeniaca*, and subgenus *Prunus*, exhibiting closer affinities. We corroborated that *P. tianshanica*, *P. dictyoneura*, and *P. humilis* within subgenus *Microcerasus* formed a separate branch situated between subgenus *Prunus* and subgenus *Armeniaca*. Additionally, we identified *P. cerasifera* and *P. divaricata* as a branch within subgenus *Prunus*, closely related to *P. domestica*. Geng et al. [23] performed chloroplast genome assembly of *P. domestica* to construct a phylogenetic tree, revealing that *P. domestica* formed a branch with *P. salicina*. Nevertheless, the chloroplast genome sequences of *P*. *divaricata* and *P. cerasifera* were not included in their phylogenetic analysis.

In the present study, we found that species of the genus *Prunus* differentiated approximately 64.93 million years ago (Mya) (95% HPD: 56.98–69.45 Mya), which aligns with the timing of the emergence of species of the genus *Prunus* [59]. Additionally, a resequencing-based study conducted by Groppi et al. on the genetic diversity and evolution of *P. armeniaca* and its wild relatives indicated a gradual divergence from *P. mume* populations to *P. armeniaca* populations. Our findings also revealed that the divergence between *P. mume* and *P. zhengheensis* occurred earlier than that between *P. armeniaca* and *P. mandshurica* [60]. Notably, *P. armeniaca* and *P. mandshurica* are more closely related, with a differentiation time of 2.46 Mya (95% HPD: 0.44–7.87 Mya).

## 5. Conclusions

The four wild *Prunus* species had the chloroplast genomes that showed a circular tetrameric structure with lengths ranging from 157,395 to 158,090 bp in length and genome lengths from those of the four species in the wild. These genomes contained a total of 130 to 131 annotated genes, comprising 85 to 86 protein-coding genes, 8 rRNA genes, and 35 to 36 tRNA genes. The chloroplast genomes across the four species displayed conservation, although *P. domestica* exhibited a deletion of the *rsp19* gene at the IRa/LSC junction. A total of 300 SSRs and 166 long repetitive sequences were identified among the four wild *Prunus* species. The variation sites were mostly found in the non-coding areas, and seven very variable regions were found. The phylogenetic analysis revealed five distinct branches, which included subg. *Armeniaca*, subg. *Microcerasus*, subg. *Prunus*, subg. *Amygdalus*, and subg. *Cerasus*. It is estimated that the crown group of *Prunus* species had a differentiation period of roughly 61.41 Mya.

## Figures and Tables

**Table 1:** Chloroplast genome features of four wild species of the genus *Prunus*.

Items		*P. tianshanica*	*P. divaricata*	*P. armeniaca*	*P. domestica*
Total Size (bp)		158,061	157,886	158,090	157,395
LSC (bp)		86,279	86,224	86,314	85,744
SSC (bp)		19,018	18,970	19,000	18,949
IR (bp)		26,382	26,346	26,388	26,351
GC (%)	Total (%)	36.73%	36.74%	36.72%	36.76%
	LSC (%)	34.55%	34.53%	34.54%	34.56%
	SSC (%)	30.44%	30.45%	30.42%	30.47%
	IR (%)	42.56%	42.63%	42.57%	42.59%
Gene number		131	131	131	130
PCG		86	86	86	85
tRNA		37	37	37	37
rRNA		8	8	8	8

Note: large single copy—LSC; small single copy—SSC; inverted repeat sequence—IR; guanine/cytosine content—GC; protein-coding gene—PCG.

**Table 2:** Chloroplast genome gene lists of four wild *Prunus* species.

Category	Group of Genes	Name of Genes
Photosynthesis	Subunits of photosystem I	*psaA*, *psaB*, *psaC*, *psaI*, *psaJ*
	Subunits of photosystem II	*psbA*, *psbB*, *psbC*, *psbD*, *psbE*, *psbF*, *psbH*, *psbI*, *psbJ*, *psbK*, *psbL*, *psbM*, *psbN*, *psbT*, *psbZ*
	Subunits of NADH dehydrogenase	ndhA*, ndhB*(2), *ndhC*, *ndhD*, *ndhE*, *ndhF*, *ndhG*, *ndhH*, *ndhI*, *ndhJ*, *ndhK*
	Subunits of cytochrome b/f complex	*petA*, petB*, petD*, *petG*, *petL*, *petN*
	Subunits of ATP synthase	*atpA*, *atpB*, *atpE*, atpF*, *atpH*, *atpI*
	Large subunit of rubisco	*rbcL*
	Subunits of protochlorophyllide reductase	-
Self-replication	Proteins of large ribosomal subunit	*rpl14*, rpl16*, rpl2*(2), *rpl20*, *rpl22*, *rpl23*(2), *rpl32*, *rpl33*, *rpl36*
	Proteins of small ribosomal subunit	*#rps19*^123^, *rps11*, rps12**(2), r*ps14*, *rps15*, rps16*, *rps18*, *rps19*, *rps2*, *rps3*, *rps4*, *rps7*(2), *rps8*
	Subunits of RNA polymerase	*rpoA*, *rpoB*, rpoC1*, *rpoC2*
	Ribosomal RNAs	*rrn16*(2), *rrn23*(2), *rrn4.5*(2), *rrn5*(2)
	Transfer RNAs	trnA-UGC*(2), *trnC-GCA*, *trnD-GUC*, *trnE-UUC*, *trnF-GAA*, *trnG-GCC*, trnG-GCC*, *trnH-GUG*, *trnI-CAU*(2), trnI-GAU*(2), trnK-UUU*, *trnL-CAA*(2), trnL-UAA*, *trnL-UAG*, *trnM-CAU*, *trnN-GUU*(2), *trnP-UGG*, *trnQ-UUG*, *trnR-ACG*(2), *trnR-UCU*, *trnS-GCU*, *trnS-GCU^23^*, *trnS-GGA^14^*, *trnS-UGA*, *trnT-GGU*, *trnT-UGU*, *trnV-GAC*(2), trnV-UAC*, *trnW-CCA*, *trnY-GUA*, *trnfM-CAU*
Other genes	Maturase	*matK*
	Protease	clpP**
	Envelope membrane protein	*cemA*
	Acetyl-CoA carboxylase	*accD*
	c-type cytochrome synthesis gene	*ccsA*
	Translation initiation factor	-
Genes of unknown function	Conserved hypothetical Chloroplast ORF	#*ycf1^3^*, *ycf1^124^*, *ycf1*, *ycf2*(2), ycf3**, *ycf4*

Gene*—gene with one intron; Gene**—gene with two introns; #Gene—pseudo gene; Gene(2)—number of copies of multi-copy genes; Gene^123^—*P. armeniaca*, *P. divaricate*, and *P. tianshanica* gene; Gene^23^—*P. divaricata* and *P. tianshanica* gene; Gene^14^—*P. armeniaca* gene and *P. domestica* gene; Gene^3^—*P. tianshanica* gene; Gene^124^—*P. armeniaca*, *P. divaricate*, and *P. domestica* gene.

## Data Availability

The assembled chloroplast genomes of *Prunus armeniaca*, *Prunus divaricata*, *Prunus tianshanica* were deposited in GenBank with the accession numbers PV138220, PV138221, PV138221.

## Supplementary Materials

The following supporting information can be downloaded at: https://www.mdpi.com/article/10.3390/genes16030239/s1, Table S1: Shows the accession numbers and species information of the chloroplast genomes that were used in the phylogenetic analysis. Table S2: Shows the relative use of four wild species of the genus *Prunus* chloroplast genomes in terms of synonymous codon. Figure S1: Construction of a phylogenetic tree of the species of the genus *Prunus* based on the ML (A) and BI (B) methods. (A) The values of the nodes in the graph are the posterior probabilities values; (B) the node values in the figure are bootstrap support values.

## Abbreviations

LSC	Large single copy
SSC	Small single copy
IR	Inverted repeat sequence
GC	Guanine/cytosine content
PCG	Protein-coding gene
SSRs	Simple sequence repeats
BI	Bayesian inference
ML	Maximum likelihood
HPD	Highest posterior density
Mya	Million years ago
