# Supplementary material for: Comparative and Phylogenetic Analysis of the Chloroplast Genomes of Four Wild Species of the Genus Prunus
Source: Genes (Basel). 2025 Feb 20;16(3):239. doi: 10.3390/genes16030239 (PMC11942447; doi:10.3390/genes16030239)
Supplement: Supplementary file 1 [file genes-16-00239-s001.zip › Supplementary table S1.pdf]

Table S1 Species information and accession numbers of the chloroplast genomes used in the phylogenetic analysis.

S.No	Species	Accession
1	*Prunus mume*	MW759300.1
2	*Prunus zhengheensis*	NC_062793.1
3	*Prunus mandshurica*	NC_068703.1
4	*Prunus tomentosa*	MK911759.1
5	*Prunus humilis*	NC_035880.1
6	*Prunus dictyoneura*	NC_051894.1
7	*Prunus cerasifera*	MZ128910.1
8	*Prunus domestica*	NC_050959.1
9	*Prunus salicina*	NC_047442.1
10	*Prunus simonii*	MW406463.1
11	*Prunus davidiana*	NC_039735.1
12	*Prunus persica*	OL449945.1
13	*Prunus pseudocerasus*	NC_030599.1
14	*Prunus pusilliflora*	OR687397.1
15	*Prunus serrulata*	OP611546.1
16	*Prunus avium*	MK622380.1
17	*Prunus cerasus*	NC_066420.1
18	*Prunus fruticosa*	NC_054254.1
19	*Malus sieversii*	NC_042192.1
20	*Crataegus songarica*	PQ149019.1
21	*Prunus ussuriensis*	NC_065730.1
