# Supplementary material for: Comparative and Phylogenetic Analysis of the Chloroplast Genomes of Four Wild Species of the Genus Prunus
Source: Genes (Basel). 2025 Feb 20;16(3):239. doi: 10.3390/genes16030239 (PMC11942447; doi:10.3390/genes16030239)
Supplement: Supplementary file 1 [file Supplementary figure S1.pdf › Supplementary figure S1.pdf]

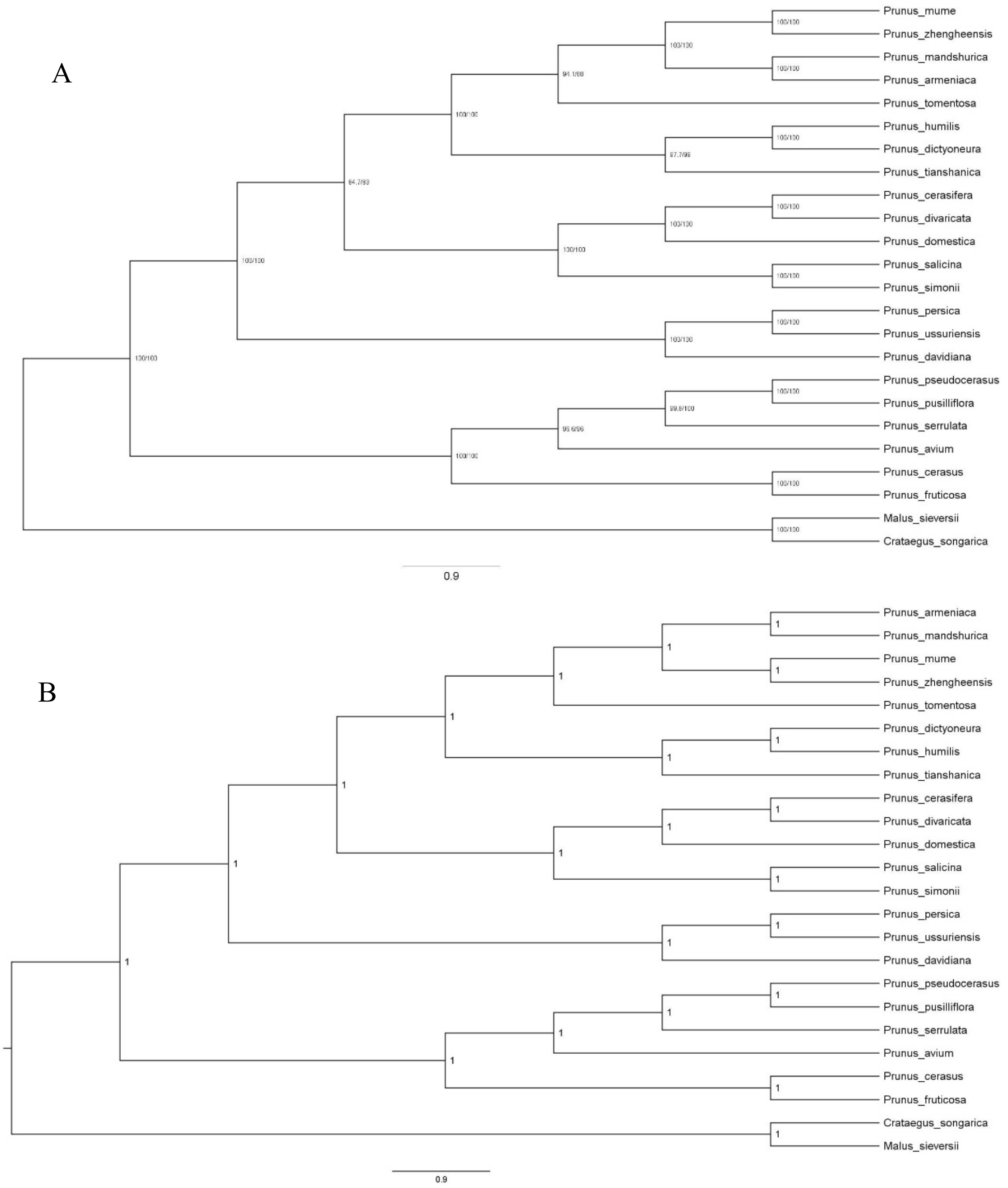

Figure S1. Construction of a phylogenetic tree of the *Prunus* spp. based on the maximum likelihood (A) and Bayesian (B) methods. A, The values of the nodes in the graph are the posterior probabilities values; B, the node values in the figure are bootstrap support values.
